# Conjunctival Autograft With Fibrin Glue for Pterygium: A Long Term Recurrence Assessment

**Published:** 2019-10-01

**Authors:** Pablo Luis Daponte, Andrea Cigna, Ovidio Lescano, Federico Sipowicz, Brenda Peña, Gabriel Abud, Gabriel Di-Virgilio, Adriana Chirinos, Gustavo Federico Bodino

**Affiliations:** 1 Centro Integral de Salud Visual Dr. Daponte, Buenos Aires, Argentina

**Keywords:** Pterygium, Sutureless, Recurrence, Fibrin Tissue Adhesive, Conjunctival Autograft, Fibrin Glue

## Abstract

Pterygium is an old challenge for ophthalmic surgeons. Its final resolution is surgical intervention. New surgical techniques have been introduced to improve the outcome, however, the possibility of recurrence always exists. The purpose of this study was to evaluate the pterygium recurrence rate with a long-term follow-up, after surgery was performed with conjunctival autograft and fibrin glue as a biological adhesive. A retrospective case-series study was performed, reviewing cases operated from May 2008 to May 2018 with at least 1 year of follow-up in a private clinic in Buenos Aires, Argentina. The evaluation time-points were at 1 day, 20 days, 6 months, 1 year after surgery and then every year. All the procedures were performed by the same surgeon in single center. Topical Mitomycin C (MMC), 5-Fluorouracil (5-FU), cauterization and/or amniotic membrane were not used in any case. From a total of 159 operated eyes (82/77 women/men), pterygium was recurred in 7 eyes (4.4%); all of them detected at the second follow-up time-point (at day 20). Intraoperative complications did not occur, but at the postoperative stage, one case presented a conjunctival granuloma, which was surgically resolved. In conclusion, a low pterygium recurrence rate was observed after conjunctival autograft with fibrin glue. In our study, recurrence was found at the postoperative first month and did not recur until the end of follow-up for 10 years.

## INTRODUCTION

The cornea must be clear to let the light get into the eye and the visual process begins. When the cornea loses its transparency, the sight could be severely affected, as happens when pterygium grows and covers the pupillary axis [1-3]. Patients with pterygium consult physicians because of discomfort, ocular surface inflammation, red eyes, (aesthetic reasons) or finally, when their vision is decreased [4, 5]. Different hypotheses exist about the pterygium pathogenesis and its progression [6-9]. However, the final decision is surgery. It seems to be easy, but usually its complexity is underestimated. Patients feel pain during and after the procedure, the conjunctiva bleeds and with traditional techniques, several conjunctival sutures must be performed and the most frustrating situation is when the pterygium recurs. Pterygium is an old challenge for ophthalmic surgeons, therefore, new surgical techniques have been introduced to improve its result, however, the possibility of recurrence always exists [5, 10-15]. To reduce recurrence rates with fewer complications, pterygium excision performed with conjunctival autografting seems to be the best surgical procedure [5, 12, 13]. Actually, the possibility to perform a sutureless surgery using biological adhesive, decreases the operation time and increases patient satisfaction (less pain) [14-21]. Here we aimed to evaluate the pterygium recurrence rate with a long term follow-up, after the operation was performed with conjunctival autograft and fibrin glue.

## METHODS

**Study Design**

A retrospective case-series study was performed, reviewing the clinical charts of patients operated due to pterygium from May 2008 to May 2018. The surgical technique was conjunctival autograft with fibrin glue performed by the same surgeon (P.L.D.) in a private clinic in Buenos Aires City, Argentina. Topical Mitomycin C (MMC), 5-Fluorouracil (5-FU), cauterization, and/or amniotic membrane were not used in any case. Exclusion criteria were those with less than one year of follow-up.

The study was conducted in accordance with the Declaration of Helsinki and an approval was obtained from the “Centro Integral de Salud Visual” Institutional Review Board/Ethics Committee. Patients were informed about surgical characteristics and their possible risks and complications. A written informed consent was obtained from all participants. 

**Parameters to Evaluate**

Baseline population data (age, gender, and the pterygium grade), intra-surgical or post-surgical complications, pterygium recurrence rate and years of follow-up were evaluated. 

Pterygium was subjectively graded by slit-lamp (Haag-Streit BI 900; The USA) always by the same observer as well as the recurrence rate (defined as any kind of fibrous-vascular tissue detected over the limbus, invading the cornea). Pterygium was originally graded in four stages, based on similar investigations [22, 23] according to the presence of fibrous-vascular tissue, from nasal limbus to the visual axis, as follows; grade 1 (less than 2.0 mm), grade 2 (higher that 2.0 mm and less than 4.0 mm), grade 3 (higher than 4.00 mm, without covering the visual axis) and grade 4 (tissue covering the visual axis). 

Routine follow-up time was performed at day 1, day 20, month 6 and the first year after the operation and then annually till 10 years. 

**Figure 1:**
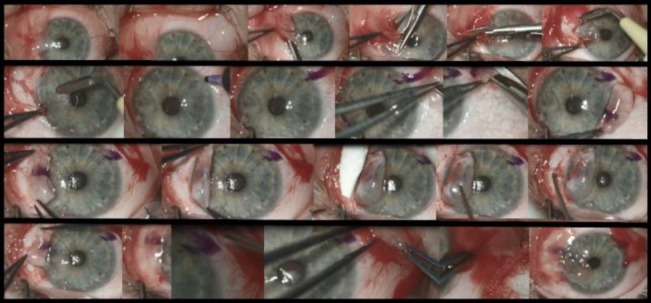
Video Frames Showing Different Steps Necessary to Excise the Pterygium Using Conjunctival Autograft and Fibrin Glue

Surgical Technique (Fig. 1): Topical anesthesia was performed with proparacaine 0.5% (Anestalcon®; Alcon) and local injection in the head of the pterygium with lidocaine/epinephrine preservative free. Firstly, for resection of the pterygium body, Westcott scissors was used and after that, the fibrovascular corneal tissue was resected with crescent, performing a keratotomy to completely remove the corneal scarring tissue. The tenon was carefully excised and the tissue was remitted to posterior anatomopathological evaluation. Then, the lower-temporal part of the conjunctiva was marked, to obtain the conjunctival graft, resecting the required size of tissue to cover the previous excised area. It was measured by compass, avoiding to excise the tenon and leaving a distance of 2 mm from the sclerocorneal limbus. The extracted tissue was everted and placed over the cornea and one of the two components from the fibrin adhesive was added all over the conjunctival graft surface. The receptor zone was dried with a Weck-cel. The second component of the fibrin adhesive was added over the bare sclera using two 0.12 forceps, the conjunctival graft tissue was moved to perform a “bed sheet maneuver”, placing the graft over the scleral bed. Both conjunctival sides should be placed correctly (one of those belong to the limbus side) and completely covering the scleral defect. One minute is required to wait until both tissues stick together completely. Then, the patient was asked to blink several times to confirm the graft adherence. Finally, dexamethasone 0.1%/tobramicine 0.3% (Tobradex® Novartis) topical ointment were administered.

The following treatment was indicated: Mixture of gatifloxacin0.3%/prednisolone acetate 1.0% (Zypred®, Allergan), every 6 hours, starting one day before the operation and then, every 2 hours until two days and after that every 6 hours during the next 20 days. Ketorolac 0.5% topical drops (Acular®, Allergan), every 8 hours, starting one day before and after the operation for 20 days. 

Topical Mitomycin C (MMC), 5-Fluorouracil (5-FU), cauterization, and/or amniotic membrane were not used in any case. The adhesive is a commercial substance (Beriplast P combi set®; CSL Behring) composed by human fibrin, human clotting factor XIII, thrombin, aprotinin and calcium chlorure, pasteurized.

Data was collected in Spreadsheets file (Google; https://www.google.com/sheets/about/) and descriptive statistics including frequency and percentage were used to present the results.

## RESULTS

A total of 159 eyes operated were included (82/77 women/men). Mean ± standard deviation (SD) of age was 53.7 ±9.1 years. All of the cases achieved at least one year of follow-up. Only 3 cases were operated due to recurrent pterygium (which no recurrence later); the rest of operations were due to primary pterygium. Pterygium was located on the nasal side in all the cases. Follow-up time and participants are presented in Table 1.

The pterygium grade and the percentage are shown in Table 2. Most cases 93 (58.5%) had grade 2 of pterygium followed by grade 3 in 46 (28.9%) cases, grade 1 in 12 (7.5%) and grade 4 in 8 (5%). Pterygium recurred in 7 eyes (4.4%); 4 (2.5%) eyes had pterygium of grade 2 and 3 (1.9%) grade 3; all of them detected at the second follow-up time (day 20). Intraoperative complications did not occur postoperatively, except for one case who presented a conjunctival granuloma, which was surgically resolved.

**Table 1:** Number of Cases and the Years of Follow-up

Years of follow-up	Number of Cases (Percentage)
1	11 (6.9)
2	20 (12.6)
3	35 (22)
4	22 (13.8)
5	15 (9.4)
6	12 (7.5)
7	10 (6.3)
8	14 (8.9)
9	15 (9.5)
10	5 (3.1)
Total of cases	**159 (100)**

## DISCUSSION

The present study showed a low recurrence after pterygium surgery using conjunctival autograft with fibrin glue in long term follow-up. Usually, the complexity of pterygium surgery is underestimated. Therefore, new surgical techniques for pterygium have been introduced to improve its outcome. However, the possibility of recurrence is always present. Kaufman SC et al. in 2013 indicated that bare sclera excision of pterygium results in a higher recurrence rate than excision accompanied by the use of certain adjuvants [24]. After that, the use of amniotic membrane seemed to be a good choice, but Clearfield E et al. compared the safety and effectiveness of conjunctival autograft compared to amniotic membrane graft and concluded that the pterygium excision with conjunctival autograft was associated with a lower risk of recurrence at 6 months [25]. A similar conclusion has been indicated by different authors [18-21, 24, 25]. The results of this study also confirm their findings.

**Table 2:** Pterygium Grade and the Recurrence Number Regarding Each Grade and the Percentage of Recurrence Rate

Pterygium grade	Eyes n (%)	Recurrence n (%)
Grade 1	12 (7.5)	0
Grade 2	93 (58.5)	4 (2.5)
Grade 3	46 (28.9)	3 (1.9)
Grade 4	8 (5)	0
Total of eyes	159 (100)	7 (4.4)

Abbreviations: n: number; %: percentage

Pterygium excision with conjunctival autografting reduces recurrence rates with fewer complications, and subsequently, has become the first choice of surgical procedure. However, a considerable number of conjunctival sutures must be performed increasing the operation time and postoperative discomfort, which are some arguments against this technique. Also, the use of sutures in pterygium surgery is associated with postoperative inflammation, discomfort and complications related to the sutures themselves [5, 18-21].

Since 1944, biological adhesives have been emerged for different use in medicine and ophthalmology [16, 17, 24-28]. For pterygium surgery, their cost could be a limitation, however, Gong J et al. compared self-made (low-cost) cryopreservative fibrin glue and commercial fibrin glue kit in pterygium surgery, concluding that both are equally effective [29]. This possibility is interesting, especially for countries with economical limitations, but unfortunately, it was not evaluated in this series. In the present work the biological adhesive used was a commercial kit. Most commonly reported complications associated to biological adhesive for pterygium surgery in the literature include graft dehiscence, graft retraction and granuloma [16, 17, 19, 28-31]. In the present series, only one case developed conjunctival granuloma which is a low rate associated with fibrin glue. 

The strong point of this study was its long term follow-up. Also, the results showed that pterygium recurrence has occurred at early stages and all of our cases detected at the second follow-up time (at day 20). If recurrence did not occur during the first postoperative month, it did not appear later (or up to ten years follow-up) in the present series. This is different from other published studies, where pterygium recurrence was detected between 6 to 12 months after surgery, using similar surgical techniques [15, 21, 22, 30].

One of the limitations of the present work was that all the operations were performed by the same surgeon with more than 30 years of surgical practice. Possibly, a learning curve would be necessary to develop this technique safety, as could happen with any other surgical procedure and good results in part could be associated with that [29].

Nevertheless, the scientific evidence reported here, along 10 years of follow up, shows that this surgical method seems to be the most efficient to avoid recurrence after pterygium surgery. Cauterization and classical adjuvant drugs as 5-FU or MMC usually were used to control wound healing and decrease recurrences [14]. Nevertheless, secondary to these substances and procedures, severe complications could appear [32]. herefore, none of these methods was used in the present study. This aspect is another issue to remark that the results in this series are only associated to the surgical technique. New studies are focused on new adjuvants therapies, principally with anti-VEGF effect, and some results seem to be promising [32-34].

It could be interesting to compare the present technique with and without anti-VEGF treatment. 

## CONCLUSIONS

In this work, a low recurrence rate after pterygium surgery was observed using conjunctival autograft and biological adhesive in ten years of follow-up. All the recurrences in this study appeared at the postoperative first month. There are other aspects not evaluated in this study, as patient comfort and surgical time, which are probably in favor of this technique as well. Finally, multicentric prospective studies could confirm the present promising results.

## DISCLOSURE

Ethical issues have been completely observed by the authors. All named authors meet the International Committee of Medical Journal Editors (ICMJE) criteria for authorship of this manuscript, take responsibility for the integrity of the work as a whole, and have given final approval for the version to be published. No conflict of interest has been presented.

## Funding/Support

None.
